# Epstein-Barr virus infection and variants of Epstein-Barr nuclear antigen-1 in synovial　tissues of rheumatoid arthritis

**DOI:** 10.1371/journal.pone.0208957

**Published:** 2018-12-11

**Authors:** Shotaro Masuoka, Natsuko Kusunoki, Ryo Takamatsu, Hiroshi Takahashi, Kazuaki Tsuchiya, Shinichi Kawai, Toshihiro Nanki

**Affiliations:** 1 Department of Internal Medicine, Toho University Graduate School of Medicine, Tokyo, Japan; 2 Division of Rheumatology, Department of Internal Medicine, Toho University School of Medicine, Tokyo, Japan; 3 Department of Inflammation and Pain Control Research, Toho University School of Medicine, Tokyo, Japan; 4 Department of Orthopedic Surgery, Toho University School of Medicine, Tokyo, Japan; Keio University, JAPAN

## Abstract

**Objective:**

The objective of the present study was to investigate Epstein-Barr virus (EBV) infection as an environmental factor for the development of rheumatoid arthritis (RA).

**Methods:**

Synovial tissues were collected during surgery from 128 RA and 98 osteoarthritis (OA) patients. DNA was extracted from synovial tissues. The EBV gene was assessed by nested PCR for the amplification of EBV nuclear antigen-1 (EBNA-1). The nucleotide sequence of the PCR product was elucidated. HLA-DRB1 genotyping was also performed.

**Results:**

EBV DNA was more frequently detected in the synovial tissues of RA patients (32.8%) than OA patients (15.3%) (p<0.01). The frequency of EBNA-1 variants did not significantly differ between RA and OA (RA: 17%, OA: 13%). The population with the HLA-DRB1 shared epitope (SE) was significantly higher in RA patients (70.3%) than in OA patients (44.9%) (p<0.001). In RA patients, the presence of EBV DNA was similar among SE-positive and -negative patients (SE-positive: 34.4%, -negative: 28.9%). The population with the EBNA-1 variant did not significantly differ between SE-positive and -negative patients (SE-positive: 12.9%, -negative: 27.3%).

**Discussion:**

The present results indicate that EBV infection contributes to the onset of RA and chronic inflammation in synovial tissues. The frequency of EBNA-1 gene variants was low and not significantly different between RA and OA, suggesting that EBNA-1 gene variants are not a risk factor for RA. HLA-DRB1 with SE is a genetic risk factor for the development of RA. However, neither the presence of EBV nor EBNA-1 gene variants differed between SE-positive and -negative RA patients. Therefore, these two risk factors, SE and EBV, may be independent.

**Conclusion:**

EBV infection may be an environmental risk factor for the development of RA, while nucleotide variants of EBNA-1 do not appear to contribute to its development.

## Introduction

Rheumatoid arthritis (RA) is characterized by chronic inflammation in multiple joints. Although the pathogenesis of RA has not yet been elucidated in detail, it appears to be a multifactorial disease involving genetic and environmental factors [1]. Among genetic factors, human leukocyte antigen DRB1 (HLA-DRB1) alleles have been shown to influence susceptibility to and the severity of RA. HLA-DRB1 alleles containing the motifs of QKRAA, QRRAA, and RRRAA at positions 70–74 of the third hypervariable region are strongly associated with RA, and these alleles have been named shared epitopes (SE). Cigarette smoking is an environmental risk factor for the development of RA; a meta-analysis showed that the incidence of RA was 26% higher in smokers than in non-smokers [2]. Microbial agents, particularly periodontal infections, are also suspected to be an environmental trigger for RA [3]. A relationship has been widely shown between Epstein-Barr virus (EBV) and RA. The first indication to a relationship between EBV and RA was reported in 1978, showing that antibodies against EBV nuclear antigen (EBNA) differ between RA patients and normal controls [4]. Tosato *et al*.[5]found that EBV-specific suppressor T-cell function was defected in RA patients, suggesting that EBV persistence with a specific regulatory T-cell deficiency may contribute to several immune abnormalities in RA. EBV DNA load in peripheral blood lymphocytes was increased in RA compared with healthy controls [6]. Antibodies against a glycine/alanine-rich repeat in EBNA-1 are cross-reactive with a 62-kDa protein present in the synovial membrane of RA, but not in that of controls [7]. Several studies have shown that latent membrane protein-1 (LMP-1) as well as EBNA-2 and Epstein–Barr virus-encoded small RNAs (EBER) could be detected in the synovial tissue in patients with RA [8–13]. Furthermore, Takei *et al*. showed that mRNA expression of EBV-specific cytotoxic T-cell-associated molecule signaling lymphocytic-activation molecule-associated protein (SAP) was decreased in the peripheral leukocytes or T cells, which may lead to failure of the immune system to eliminate EBV in RA patients [14]. Interestingly, inoculation with EBV developed erosive arthritis in mice with humanized immune system [15]. Moreover, a large proportion of anti-citrullinated protein/peptides antibody-producing plasma cells surrounding synovial germinal centers were infected with EBV [16].

EBV is also considered to be a trigger for the development of several malignancies, such as various lymphomas, nasopharyngeal carcinoma, and gastric carcinoma. Previous studies reported several nucleotide variants of EBNA-1, and mutated EBNA-1 has frequently been detected in nasopharyngeal carcinoma [17]. Therefore, EBNA-1 variants may contribute to the development of malignancy. It has never been examined whether the mutation of EBNA-1 contributes to the pathology of RA.

In the present study, we analyzed the presence of EBV as well as the variants of EBNA-1 in the synovial tissues of RA patients.

## Patients and methods

### Patients and tissue specimens

RA and osteoarthritis (OA) tissue specimens were obtained from patients undergoing total knee replacement surgery who fulfilled the American College of Rheumatology criteria for RA [18]. The study protocol was approved by the Ethics Committees of Faculty of Medicine, Toho University (approval numbers, A17016_A16007), and all patients gave consent for the use of their tissue in the present study. One hundred and twenty-eight RA patients and 98 OA patients have been included in the present study.

Synovial tissues were obtained under sterile conditions, frozen immediately in liquid nitrogen, and then stored at -80°C until used. All tissues were unlinkable and anonymized, and, thus, it was not possible to refer clinical data to any case.

### DNA extraction

Frozen synovial tissue samples were left to stand at room temperature for 30 minutes. DNA was extracted by the QIAamp DNA mini kit (Qiagen, GmbH, Hilden, Germany) according to the manufacturer’s instructions.

### PCR amplification and DNA sequencing

The extracted genomic DNA (100 μg) was amplified using 0.5 μl of each 50 mM primers in 50 μl volume containing 25 mM MgCl_2_, 1.25 units of HotStarTaq DNA polymerase (Qiagen, GmbH), and 10 mM of each dNTP (Qiagen, GmbH). PCR was performed with the β-globin-specific primers 5'-ACA CAA CTG TGT TCA CTA GC-3' and 5'-TGG TCT CCT TAA ACC TGT CTT G-3' using a thermal cycler (TaKaRa Biomedicals, Ohtsu, Shiga, Japan) programmed at 95°C for 15 minutes to activate HotStarTaq DNA polymerase followed by 30 cycles at 94°C for 1 minute for denaturation, at 55°C for 2 minutes for annealing, at 72°C for 1 minute for extension, and at 68°C for 7 minutes for final extension [19]. To amplify the EBNA-1 gene, EBNA-1-specific primers (5'-AGA TGG TGA GCC TGA CGT G-3' and 5'-GCA TCC TTC AAA ACC TCA GC-3') were used with the following program: at 95°C for 15 minutes to activate HotStarTaq DNA polymerase followed by 35 cycles at 94°C for 45 seconds for denaturation, at 60°C for 45 seconds for annealing, at 68°C for 45 seconds for extension, and at 68°C for 7 minutes for final extension. The amplicon of first PCR (2 μl) was used for second nested PCR with the nested EBNA-1-specific primers (5'-CCC GCA GAT GAC CCA GGA GA-3' and 5'-GGG TCC AGG GGC CAT TCC AAA-3') with the same PCR program as first PCR. PCR products were separated on 2% agarose gels in Tris acetate EDTA buffer stained with ethidium bromide and photographed under ultraviolet light using LAS-3000 (Fujifilm Corp, Tokyo, Japan).

To evaluate the sensitivity of the PCR, first round of PCR product was purified, and DNA copy number was calculated. One, 5, 10, 20, 50, 100 and 1000 DNA copies of the PCR product were amplified by the above PCR protocol. After the nested PCR, PCR products were detected from 5 DNA copies or more DNA copies.

The DNA nucleotide sequence of the amplified EBNA-1 gene was elucidated by Sigma-Aldrich Japan (Tokyo, Japan). Data from the nucleotide sequence was analyzed using BLAST (National Center for Biotechnology Information) and compared with the B95-8 strain [20]. Alignments between sequences were analyzed using DNADynamo (Blue Tractor Software, North Wales, UK).

### HLA-DRB1 genotyping

HLA-DRB1 genotyping was performed using extracted genomic DNA from synovial tissue by ReproCELL (Kanagawa, Japan).

### Statistical analysis

Statistical analyses were performed with Prism ver. 5.0 software (Graphpad Software, San Diego, CA). Patients with RA and OA were compared with Fisher’s exact test. A p value less than or equal to 0.01 was considered to be significant.

## Results

Synovial tissue samples were collected from 128 RA and 98 OA patients. The β-globin gene was detected by PCR in all genomic DNA extracted from the synovial tissues of RA and OA. Using synovial tissues, the EBNA-1 gene was amplified by PCR. The EBNA-1 gene was detected in 42 out of 128 RA samples (32.8%) (Table 1), but in only 15 out of 98 OA samples (15.3%). The frequency of EBNA-1 DNA detection was significantly higher in RA than in OA samples.

**Table 1:** Frequency of the presence of EBNA-1 DNA in synovial tissues of RA and OA patients.

	No. (%) in RA (n = 128)	No. (%) in OA (n = 98)	P value
**EBNA-1-positive**	42 (32.8)	15 (15.3)	<0.01

EBNA-1, Epstein-Barr virus nuclear antigen-1; RA, rheumatoid arthritis; OA, osteoarthritis.

Several variants of the EBNA-1 gene were previously reported, particularly at amino acid residue 487 [21]. We analyzed nucleotide sequences across amino acid residues from 468 to 533 of EBNA-1 PCR products. In a previous study, the EBNA-1 nucleotide sequence was examined using 8 lymphoid tissues from Japanese individuals living in Japan, and all were found to have an identical EBNA-1 nucleotide sequence [22], which was considered to be the Japanese prototype. We compared analyzed nucleotide sequences with the Japanese prototype sequence. Most of the EBNA-1 gene sequences were identical to the Japanese prototype sequence [35 out of 42 (83.3%) in RA, 13 out of 15 (86.7%) in OA] (Table 2). A variant [Variant 1: CCG (proline) to CAG (glutamine) at amino acid residue 476, GTT (valine) to ACT (threonine) at 487, AGT (serine) to TGT (cysteine) at 492, and GAG (glutamic acid) to GAT (aspartic acid) at 499] was found in 5 RA (11.9%) and 1 OA (6.7%). Furthermore, other variants [Variant 2: CAA (glutamine) to CAC (histidine) at 471, CCG (proline) to CAG (glutamine) at 476, GTT (valine) to ACT (threonine) at 487, AGT (serine) to TGT (cysteine) at 492, and GAA (glutamic acid) to GAT (aspartic acid) at 500] and [Variant 3: GAA (glutamic acid) to GAC (aspartic acid) at 483] were found in 1 RA (2.4%). A variant [Variant 4: GTT (valine) to GCT (alanine) at 487, and GAA (glutamic acid) to TTA (leucine) at 500] was also found in 1 OA (6.7%). The population of the Japanese prototype or variants of the EBNA-1 gene was not significantly different between RA and OA samples.

**Table 2:** Nucleotide and amino acid sequences of EBNA-1 in synovial tissues of RA and OA patients.

No. of amino acid residues	471	476	483	487	492	499	500
**No. (%) in RA (n = 42)**							
**Japanese prototype [35 (83.3%)]**	CAA (Q)	CCG (P)	GAA (E)	GTT (V)	AGT (S)	GAG (E)	GAA (E)
**Variant 1** **[5 (11.9%)]**		CAG (Q)		ACT (T)	TGT (C)	GAT (D)	
**Variant 2** **[1 (2.4%)]**	CAC (H)	CAG (Q)		ACT (T)	TGT (C)		GAT (D)
**Variant 3** **[1 (2.4%)]**			GAC (D)				
**No. (%) in OA (n = 15)**							
**Japanese prototype [13 (86.7%)]**	CAA (Q)	CCG (P)	GAA (E)	GTT (V)	AGT (S)	GAG (E)	GAA (E)
**Variant 1** **[1 (6.7%)]**		CAG (Q)		ACT (T)	TGT (C)	GAT (D)	
**Variant 4** **[1 (6.7%)]**				GCT (A)			TTA (L)

The nucleotide sequence of amplified EBNA-1 DNA in synovial tissue was analyzed. The nucleotide (amino acid) sequences of the Japanese prototype and variants (different from the Japanese prototype) were shown. EBNA-1, Epstein-Barr virus nuclear antigen-1; RA, rheumatoid arthritis; OA, osteoarthritis; Q, glutamine; P, proline; E, glutamic acid; V, valine; S, serine; T, threonine; C, cysteine; D, aspartic acid; H, histidine; A, alanine; L, leucine.

A previous study reported that a subset of HLA-DRB1 alleles called SE, a five-amino acid sequence motif in residues 70–74 (QKRAA, RRRAA and QRRAA), including *0101, *0102, *0401, *0404, *0405, *0408, *0410, *1001, *1402, and *1406, was associated with the development and severity of RA [23]. We identified the HLA-DRB1 genotype in all the RA and OA samples studied, and examined the relationship between the HLA-DRB1 allele and EBV infection or a variant of EBNA-1. The frequency of SE positivity was significantly higher in RA (90 out of 128, 70.3%) than in OA samples (44 out of 98, 44.9%) (Table 3). Moreover, 2 SE alleles were more frequently observed in RA patients than in OA patients [RA: 22 out of 128 (17.2%), OA: 2 out of 98 (2.0%), p<0.001].

**Table 3:** DRB1 allele in RA and OA patients.

HLA DRB1	No. (%) in RA (n = 128)	No. (%) in OA (n = 98)	P value
**SE-positive**^a^	90 (70.3)	44 (44.9)	<0.001
*0101	19 (14.8)	9 (9.2)	NS
*0401	8 (6.3)	4 (4.1)	NS
*0404	2 (1.6)	0 (0.0)	NS
*0405	63 (49.2)	22 (22.4)	<0.001
*0410	6 (4.7)	6 (6.1)	NS
*1001	5 (3.9)	2 (2.0)	NS
*1406	6 (4.7)	3 (3.1)	NS
**2SE**	22 (17.2)	2 (2.0)	<0.001
**1SE**	68 (53.1)	42 (42.9)	NS

^a^An individual carrying at least 1 SE allele was defined as positive.

RA, rheumatoid arthritis; OA, osteoarthritis; SE, shared epitope; NS, not significant; 2SE, shared epitope with 2 alleles; 1SE, shared epitope with 1 allele.

The frequency of EBV DNA in RA synovial tissues was compared between SE-positive and -negative patients. Thirty-one out of 90 SE-positive patients (34.4%) and 11 out of 38 SE-negative RA patients (28.9%) were positive for EBV (Table 4), with no significant differences being observed in these frequencies. We also compared the frequency of positivity for EBNA-1 among SE with 2 alleles, SE with 1 allele, and SE negativity, and found no significant differences (Table 5).

**Table 4:** Relationship between the presence of EBNA-1 and SE in RA patients.

	No. (%) in SE-positive patients (n = 90)	No. (%) in SE-negative patients (n = 38)	P value
**EBNA-1-positive**	31 (34.4)	11 (28.9)	NS

EBNA-1, Epstein-Barr virus nuclear antigen-1; SE, shared epitope; RA, rheumatoid arthritis; NS, not significant

**Table 5:** Relationship between the presence of EBNA-1 and SE with 2 alleles, SE with 1 allele or SE negativity in RA patients.

	No. (%) in 2SE-positive patients (n = 22)	No. (%) in 1SE-positive patients (n = 68)	No. (%) of SE-negative patients (n = 38)	P value
**EBNA-1-positive**	5 (22.7)	26 (38.2)	11 (28.9)	NS

EBNA-1, Epstein-Barr virus nuclear antigen-1; SE, shared epitope; RA, rheumatoid arthritis; NS, not significant; 2SE, shared epitope with 2 alleles; 1SE, shared epitope with 1 allele.

We then examined the relationship between SE and EBNA-1 variants in RA samples. Four out of 31 SE-positive samples (12.9%) and 3 out of 11 SE-negative samples (27.3%) had EBNA-1 variants (Table 6). The frequencies of EBNA-1 variants did not significantly differ. Furthermore, the frequencies of EBNA-1 variants in SE with 2 alleles, SE with 1 allele, and SE negativity were not significantly different (Table 7).

**Table 6:** Relationship between EBNA-1 variants and SE in RA patients.

	No. (%) in SE-positive patients (n = 31)	No. (%) in SE-negative patients (n = 11)	P value
**EBNA-1-variant**	4 (12.9)	3 (27.3)	NS

EBNA-1, Epstein-Barr virus nuclear antigen-1; SE, shared epitope; RA, rheumatoid arthritis; NS, not significant

**Table 7:** Relationship between EBNA-1 variants and SE with 2 alleles, SE with 1 allele or SE negativity SE in RA patients.

	No. (%) in 2SE-positive patients (n = 5)	No. (%) in 1SE-positive patients (n = 26)	No. (%) of SE-negative patients (n = 11)	P value
**EBNA-1-variant**	0 (0.0)	4 (15.4)	3 (27.3)	NS

EBNA-1, Epstein-Barr virus nuclear antigen-1; SE, shared epitope; RA, rheumatoid arthritis; NS, not significant; 2SE, shared epitope with 2 alleles; 1SE, shared epitope with 1 allele

## Discussion

Previous studies reported the detection of EBV DNA in the synovial tissues of approximately 47% of RA patients and 17% of controls [10–12, 24]. Also, expressions of RNA level of EBER and protein level of LMP-1 in the RA synovial tissue were reported [8–13]. In the present study, we analyzed a large number of patients, and revealed that the population with EBV DNA in synovial tissues was larger in RA than in OA patients, which is consistent with previous findings. These results indicate that EBV infection contributes to the onset of RA and chronic inflammation in synovial tissues.

EBNA-1 is an EBV nuclear antigen that is consistently expressed in all infected cells. It is essential for the maintenance of the viral episome and is the only viral protein required for the replication of the latent form of EBV [25]. Previous studies identified a high frequency of variants at amino acid residues 466–527 in the EBNA-1 C terminus [22], and specific EBNA-1 may play a significant role in EBV-related nasopharyngeal carcinoma [17].

Therefore, we extended the present study to elucidate whether the EBNA-1 variant plays a role in the development of RA. We elucidated the nucleotide sequence of the EBNA-1 C terminus in RA synovial tissue and compared it with that in OA. The frequency of EBNA-1 variants was low and did not significantly different between RA and OA (RA: 17%, OA: 13%). The most frequently identified variant (variant 1) was detected in RA and OA. Collectively, these results suggest that EBNA-1 gene variants are not a risk factor for RA. According to the information from the manufacture, PCR error may occur 1/10,000 base pair. We found that 17% and 13% of variants in RA and OA, respectively, in analyzed 198 base pairs of the nested PCR. Therefore, most of the variants should not be caused by PCR errors.

As described above, SE in HLA-DRB1 is a genetic risk factor for RA, and we showed that EBV infection may be an environmental risk for the development of RA. Therefore, we analyzed the relationship between SE and EBV infection. However, the frequencies of EBV positivity and EBNA-1 variants were similar between SE-positive and -negative RA patients. Therefore, these two risk factors may be independent.

EBV glycoprotein gp110 contains the QKRAA amino acid sequence [26], which is homologous or similar to the SE of HLA-DRB1 molecules. Antibodies to gp110 develop during infectious mononucleosis. Therefore, B and T cells may both cross-react with the QKRAA protein sequence in gp110 and the SE of HLA-DRB1. However, the present results did not support the relationship between SE and EBV infection. EBV infection may also involve an SE-independent mechanism for the development of RA.

We further need to analyze the effect of activity of RA and treatment to the relation of RA and EBV, and also need to analyze other inflammatory arthritis, such as psoriasis, to compare RA.

In conclusion, EBV infection may be an environmental risk factor for the development of RA, while nucleotide variants of EBNA-1 do not appear to contribute to its development.
